# The Minimum Biological Energy Quantum

**DOI:** 10.3389/fmicb.2017.02019

**Published:** 2017-10-25

**Authors:** Volker Müller, Verena Hess

**Affiliations:** Department of Molecular Microbiology and Bioenergetics, Institute of Molecular Biosciences, Johann Wolfgang Goethe University, Frankfurt, Germany

**Keywords:** chemiosmosis, ATP synthesis, membrane potential, archaea, bacteria

## Abstract

Some anaerobic archaea and bacteria live on substrates that do not allow the synthesis of one mol of ATP per mol of substrate *via* substrate level phosphorylation (SLP). Energy conservation in these cases is only possible by a chemiosmotic mechanism that involves the generation of an electrochemical ion gradient across the cytoplasmic membrane that then drives ATP synthesis *via* an ATP synthase. The minimal amount of energy required for ATP synthesis is thus dependent on the magnitude of the electrochemical ion gradient, the phosphorylation potential in the cell and the ion/ATP ratio of the ATP synthase. It was always thought that the minimum biological energy quantum is defined as the amount of energy required to translocate one ion across the cytoplasmic membrane. We will discuss the thermodynamics of the reactions involved in chemiosmosis and describe the limitations for ion transport and ATP synthesis that led to the proposal that at least −20 kJ/mol are required for ATP synthesis. We will challenge this hypothesis by arguing that the enzyme energizing the membrane may translocate net less than one ion: By using a primary pump connected to an antiporter module a stoichiometry below one can be obtained, implying that the minimum biological energy quantum that sustains life is even lower than assumed to date.

## Introduction

The most important task of a cell is to divide and produce daughter cells. This requires the biosynthesis of macromolecules such as, lipids, proteins, and carbohydrates from smaller precursors. Obviously, biosynthesis requires energy that is provided by the uptake and conversion of nutrients. It was found in 1929 that anabolism and catabolism are coupled by adenosine triphosphate (ATP), which acts as an “energy currency” of every living cell (Lohmann, 1929; Lipmann, 1941). This opened the rush after the question how ATP is synthesized. Most of the experiments at that time were done with cells that degraded an organic molecule such as, glucose *via* glycolysis and it turned out that some reactions of the glycolysis pathway are directly coupled to the synthesis of ATP from ADP and inorganic phosphate (P_i_) (Bücher and Pfleiderer, 1955). This is now termed “substrate level phosphorylation” (SLP), but there are only few reactions known that energetically allow for SLP. Oxidation of the organic molecule does not only provide ATP via SLP, but also precursors for biosynthetic reactions as well as electrons or “reducing equivalents.” It is now textbook knowledge that these electrons are channeled into a membrane-bound respiratory chain, which channels electrons to a terminal respiratory enzyme that reduces the final electron acceptor. This exergonic electron flow from the donor to the acceptor is coupled to the translocation of ions across, in bacteria, the cytoplasmic membrane. Thus, the energy released from the redox reaction is “stored” as a transmembrane electrochemical ion gradient (Mitchell, 2011). This ion gradient across the membrane may then be used as a driving force for ATP synthesis, the uptake of several nutrients and motility in bacteria. An enzyme present in every domain of life, the ATP synthase, is a nanomachine driven by the transmembrane electrochemical ion gradient to produce ATP (Boyer, 1997). Peter Mitchell termed this process as “chemiosmotic mechanism of ATP synthesis,” a chemical reaction that produces an osmotic gradient across a membrane, which then drives ATP synthesis, an ingenious concept that is now textbook knowledge. Very often, the term chemiosmosis is used synonymously with “aerobic respiration“ or “electron transport phosphorylation.” Mitochondria have been the prime example to study the molecular and mechanistic principles of how electron flow leads to ion transport and these studies, now also including bacteria and archaea, have and will continue to provide exciting insights into membrane transport reactions (Schäfer et al., 1999; Mayer and Müller, 2014).

For eukaryotes respiring glucose, it is clear that chemiosmosis provides about 89% of the ATP that is generated from the oxidation of one molecule glucose to CO_2_, thus it is the most important mechanism of ATP synthesis for a living cell. Bacteria and archaea also conserve energy by SLP and chemiosmosis, but in contrast to eukaryotes, they have a huge variety of different mechanisms for chemiosmotic energy conservation, involving a broad range of diverse proteins that differ from those of mitochondria. Furthermore, the coupling ion can be Na^+^ as well as H^+^, and most astonishingly, ion transport is not necessarily driven by electron transport but also by a chemical reaction, with methyl transfer (Müller et al., 1988; Becher et al., 1992; Gottschalk and Thauer, 2001) or decarboxylating reactions (Buckel and Semmler, 1982; Dimroth, 1982) being the most prominent examples. Therefore, the term “electron transport phosphorylation” as synonym for chemiosmosis is too narrow. Instead, we should use the term “ion gradient-driven phosphorylation” (IGP) that includes the before mentioned systems. In this minireview, we will not highlight the structure and function analyses of these transport systems in bacteria and archaea, since this subject is covered intensively in the literature (Sazanov and Hinchliffe, 2006; Brandt, 2011). Instead, we will elaborate on the thermodynamic principles of energy conservation and discuss the question, how much energy is required to drive the phosphorylation of ADP. This will culminate in a proposal for the minimum biological energy quantum.

## Thermodynamics of SLP and IGP

**Figure 1:**
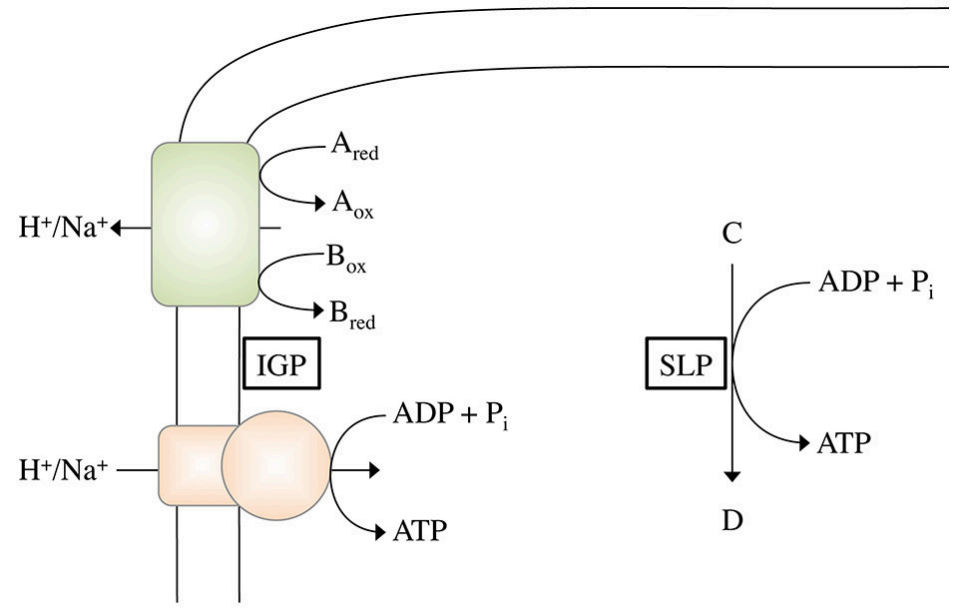
Schematic depiction of ATP synthesis by chemiosmotic ion gradient-driven phosphorylation (IGP) and substrate level phosphorylation (SLP). During IGP, electron transfer from the electron donor A_red_ to the electron acceptor B_ox_ (with the pair A_ox_/A_red_ having a more negative redox potential than the pair B_ox_/B_red_) drives the formation of an electrochemical H^+^ or Na^+^ potential across the cytoplasmic membrane, which can then be used by the ATP synthase to phosphorylate ADP. During SLP, a P_i_ group from the phosphorylated intermediate C is directly transferred to ADP.

In SLP, a chemical reaction is directly coupled to ATP synthesis (Figure 1). Thus, the reaction must liberate the amount of energy required to phosphorylate ADP:

(1) $$\begin{array}{l} ADP+P_{i}\to ATP+H_{2}O\\ \;\Delta G^{0^{\prime }}=31.8\;kJ/mol \end{array}$$

Consequently, a reaction thermodynamically able to phosphorylate ADP must have a phosphoryl group transfer potential that is more negative than −31.8 kJ/mol (Thauer et al., 1977). This restricts the reactions to only a few:

Phosphoglycerate kinase:

(2) $$\begin{array}{l} 1,3-Bisphosphoglycerate+H_{2}O\to 3-Phosphoglycerate+P_{i}\\ \;\Delta G^{0^{\prime }}=-51.9kJ/mol \end{array}$$

Pyruvate kinase:

(3) $$\begin{array}{l} Phosphoenolpyruvate+H_{2}O\to Pyruvate+P_{i}\\ \;\Delta G^{0^{\prime }}=-51.6\;kJ/mol \end{array}$$

Acetate kinase:

(4) $$\begin{array}{l} Acetylphosphate+H_{2}O\to Acetate+P_{i}\\ \;\Delta G^{0^{\prime }}=-44.8kJ/mol \end{array}$$

Arginine kinase:

(5) $$\begin{array}{l} Argininephosphate+H_{2}O\to Arginine+P_{i}\\ \;\Delta G^{0^{\prime }}=-45.0kJ/mol \end{array}$$

Carbamate kinase:

(6) $$\begin{array}{l} Carbamylphosphate+H_{2}O\to Carbamate+P_{i}\\ \;\Delta G^{0^{\prime }}=-39.3\;kJ/mol \end{array}$$

Creatine kinase:

(7) $$\begin{array}{l} Creatinephosphate+H_{2}O\to Creatine+P_{i}\\ \;\Delta G^{0^{\prime }}=-43.3kJ/mol \end{array}$$

Succinyl-CoA synthetase:

(8) $$\begin{array}{l} Succinyl-CoA\to Succinate+HS-CoA\\ \;\Delta G^{0^{\prime }}=-27.0kJ/mol \end{array}$$

Butyrate kinase:

(9) $$\begin{array}{l} Butyrylphosphate+H_{2}O\to Butyrate+P_{i}\\ \;\Delta G^{0^{\prime }}=-35.6kJ/mol \end{array}$$

Reactions shown in Equations (2–4) are widespread, whereas the others are more or less restricted to bacterial fermentation pathways.

In IGP, a membrane protein translocates an ion (either H^+^ or Na^+^) across the membrane, thereby establishing an electrochemical ion gradient (Δ$\tilde{\mu }$_Ion_). This gradient can then be used by an ATP synthase to drive the phosphorylation of ADP (Figure 1). Thus energy conservation *via* IGP includes two partial reactions, (i) the formation of the electrochemical gradient and (ii) the use of this gradient to drive ATP synthesis. Regarding the first partial reaction, the initial question is: how much energy is required to translocate one ion across the membrane? According to:

(10) $$\begin{array}{l} \Delta G=-n\times F\times \Delta {\tilde{\mu }}_{Ion} \end{array}$$

with *n* = number of ions translocated, F = Faraday constant (96.5 kJ × mol^−1^ × V^−1^), and $\Delta {\tilde{\mu }}_{Ion}$ = electrochemical ion potential,

the number of ions translocated (*n*) depends on the magnitude of the $\Delta {\tilde{\mu }}_{Ion}$. At a $\Delta {\tilde{\mu }}_{Ion}$ of −150 mV, the ΔG of the reaction has to be at least −14.5 kJ/mol in order to translocate a minimum of one ion, whereas at −210 mV, −20.2 kJ/mol are required. Accordingly, a small value for $\Delta {\tilde{\mu }}_{Ion}$ results in a lower ΔG “threshold” that is needed for the translocation of one ion. ${\tilde{\mu }}_{Ion}$ has been measured for only a few bacteria and archaea and amounts to −150 to −200 mV (Kashket, 1982; Blaut and Gottschalk, 1984; Castle et al., 1986). Notably, organisms living under extreme energy limitation such as, methanogens have comparably low $\Delta {\tilde{\mu }}_{Ion}$-values of around −150 mV (Blaut and Gottschalk, 1984; Müller et al., 1987).

The next question relates to the second partial reaction: How much energy is required to drive ATP synthesis by the ATP synthase? This value is defined by the phosphorylation potential ΔG_P_ in the cell:

(11) $$\begin{array}{l} \Delta G_{P}=-n\times F\times \Delta {\tilde{\mu }}_{Ion} \end{array}$$

with *n* = number of ions imported per every ATP that is synthesized, F = Faraday constant (96.5 kJ × mol^−1^ × V^−1^), and $\Delta {\tilde{\mu }}_{Ion}$ = electrochemical ion potential.

Under standard conditions, this value is 31.8 kJ/mol (see Equation 1). Astonishingly, the value under cellular conditions has been determined for just a few organisms: *Streptococcus lactis* (46.6 kJ/mol ATP; anaerobic cultivation on glucose; Maloney, 1983), *Escherichia coli* (48 kJ/mol ATP; aerobic cultivation on glycerol; Kashket, 1982), *Saccharomyces cerevisiae* (50 kJ/mol ATP, aerobic cultivation on glucose; Wallace et al., 1994) or for mitochondria from rat liver (64 kJ/mol ATP, aerobic respiration with succinate Slater et al., 1973). Textbooks usually refer to 50–60 kJ/mol, a range which is supported by the measured membrane potentials. Assuming a $\Delta {\tilde{\mu }}_{Ion}$ of −180 to −210 mV, this amounts to a ΔG_P_ of 69 to 81 kJ/mol. Please note that all these calculations are based on an ion/ATP stoichiometry of the ATP synthase of 4. This number is taken for the sake of clarity despite the fact that it can vary from 3 to 5, depending on the amount of ion binding sites in the *c* ring of the ATP synthase. At a given $\Delta {\tilde{\mu }}_{Ion}$, an increase of “*n*” will sustain a higher ΔG_P_, a decrease will sustain a lower ΔG_P_. If we assume the highest ion/ATP ratio detected in an ATP synthase, which is five (Pogoryelov et al., 2012), this would sustain a ΔG_P_ of 87 kJ/mol, if we assume *n* = 3.3 as in *Acetobacterium woodii* (Matthies et al., 2014), this would sustain a ΔG_P_ of only 57 kJ/mol. (For readers interested in a more detailed analysis of the impact of different *c* ring stoichiometries on the phosphorylation potential, we would like to refer to a recently published review Mayer and Müller, 2014). Unfortunately, the ΔG_P_ has been determined experimentally for only a few species, among those only one organism that lives under extreme energy limitations: *A. woodii*. This anaerobic, acetogenic bacterium uses the Wood-Ljungdahl pathway to grow on H_2_ + CO_2_, thus it uses the very same pathway for both carbon fixation and ATP synthesis. The free energy change of acetogenesis from H_2_ + CO_2_ allows for the synthesis of only a fraction of an ATP under environmental conditions and *A. woodii* clearly is a paradigm for microbial life under extreme energy limitation (Schuchmann and Müller, 2014). However, it was unknown how much energy is actually required to synthesize one ATP under these conditions. The phosphorylation potential in cells metabolizing three different acetogenic substrates (lithotrophic and organotrophic) was determined and it accounts to 37.9 ± 1.3 kJ/mol during acetogenesis from fructose, 32.1 ± 0.3 kJ/mol during acetogenesis from H_2_ + CO_2_ and 30.2 ± 0.9 kJ/mol during acetogenesis from CO, the lowest phosphorylation potential ever described (Spahn et al., 2015). Since in addition, the ion/ATP stoichiometry is known (*n* = 3.3), we can calculate the $\Delta {\tilde{\mu }}_{Ion}$ according to Equation (11) to −119 to −94 mV, the lowest value reported. Substituting these values for $\Delta {\tilde{\mu }}_{Ion}$ into Equation (10), we can now also calculate the actual amount of energy that is required to translocate one ion across the membrane: in this case, 11.5 to 9.1 kJ/mol are sufficient, due to the low $\Delta {\tilde{\mu }}_{Ion}$.

IGP evolved very early in life history and enabled the first life forms to make a living from the oxidation of gaseous compounds such as, hydrogen, carbon monoxide, or formate, coupled to the reduction of, for example, CO_2_, Fe^3+^, or S^0^ (Martin et al., 2008; Lane and Martin, 2012). The oxidation of these substrates yields only very little energy that can be harvested, too little for SLP but apparently enough for IGP. How can this be explained? For SLP, all energy required to phosphorylate one ADP has to be provided through one single reaction, which implies that the energy releasing reaction and the energy consuming reaction are strictly coupled. For IGP, however, the reaction that leads to the formation of the electrochemical gradient does not have to work with the same stoichiometry as the ATP synthase does: Assuming the ATP synthase to import four ions for the phosphorylation of one ADP, the reaction that forms the ion gradient (partial reaction 1) does not necessarily have to export four ions at one go, but might just as well proceed two times (exporting two ions per reaction) or even four times, with one ion exported each time. Thus the minimum amount of energy that is required to drive ATP synthesis *via* IGP, eventually, is the amount of energy that is initially needed to pump at least one ion across the cytoplasmic membrane. Therefore, the minimum biological energy quantum is so far regarded to be around −20 kJ/mol (Schink, 1997), the amount of energy required to pump one ion at an electrochemical membrane potential of around −200 mV. However, as discussed above, the value can be lower, and indeed *in situ* analyses suggest that growth proceeds down to −10 kJ/mol (Hoehler et al., 2001).

## A mechanistic explanation for membrane energization with low ΔG value substrates

If we assume a high $\Delta {\tilde{\mu }}_{Ion}$ of −120 mV, even with only one ion translocated we will not get down to ΔGs lower than −10 kJ/mol. But could it be possible to translocate less than one ion? Recently, we have made a proposal for *Thermococcus onnurineus*, an anaerobic hyperthermophilic archaeon isolated from a deep-sea hydrothermal vent within the PACMANUS field near the Manus Basin in the Pacific Ocean. It grows optimally at pH 8.5 and 80°C and requires complex substrates such as, yeast extract, peptone, casein, or starch (Bae et al., 2006). The outstanding feature of *T. onnurineus* is its growth on formate as carbon and energy source (Kim et al., 2010) according to:

(12) $$\begin{array}{l} HCOO^{-}+H_{2}O\to HCO_{3}^{-}+H_{2}\\ \;\Delta G^{0^{\prime }}=+1.3kJ/mol \end{array}$$

Based on the very unfavorable thermodynamics with ΔG^0^′ being positive, this reaction had been thought to enable growth in syntrophic cultures only. Amazingly, *T. onnurineus* grows by that reaction in pure culture! At a temperature of 80°C, the ΔG^0^ becomes slightly negative (−2.6 kJ/mol). This is a good example for a type of metabolism that is not possible at moderate but only at higher temperatures. By analyzing the concentrations of formate, HCO${}_{3}^{-}$ and H_2_, the ΔG of this reaction was calculated to be −8 to −20 kJ/mol, the lowest value ever reported for an organism (Kim et al., 2010). There is discussion in the field whether the cells do grow by oxidation of formate only or if the yeast extract present in the broth contributes to growth. Anyway, resting cells have been clearly shown to couple formate oxidation according to Equation (12) to the synthesis of ATP (Kim et al., 2010). Therefore, these findings question the current concept of the minimal energy value that sustains life.

How is formate oxidation coupled to energy conservation? According to molecular and genetic studies the ion-translocating electron transfer system is supposed to be rather simple. It is postulated that formate is taken up by a formate transporter and oxidized to carbon dioxide and hydrogen by a soluble formate dehydrogenase module (Fdh2), which is coupled to a membrane-bound hydrogenase module (Mfh2) (Kim et al., 2010; Mayer and Müller, 2014). Experimental data are consistent with the hypothesis that formate oxidation leads to H^+^ translocation across the cytoplasmic membrane (via the Mfh2 module) and that the H^+^ gradient is exchanged against a Na^+^ gradient via a multisubunit Na^+^/H^+^ antiporter module (Mnh2/Mrp). Please note that these membrane-bound hydrogenases are the evolutionary ancestors of complex I (NADH:quinone oxidoreductase) as found in mitochondria and bacteria (Hedderich and Forzi, 2005). The electrochemical Na^+^ gradient established then drives ATP synthesis (Lim et al., 2014). These data give a mechanistic explanation for chemiosmotic energy conservation coupled to formate oxidation to CO_2_ and H_2_.

As determined experimentally, electron flow from formate to protons allows for the generation of a transmembrane ion gradient. According to Equation (10), ΔG-values of −8 to −20 kJ for the free energy change associated with formate oxidation to CO_2_ and H_2_ (Lim et al., 2012) are sufficient to translocate 0.5–1.2 Na^+^ out of the cell, assuming a transmembrane electrochemical Na^+^ potential ($\Delta {\tilde{\mu }}_{Na^{+}}$) of −180 mV. If we assume the *c* ring to have 12 Na^+^ binding sites (thus *n* = 4), this would allow for only 0.125–0.3 mol ATP per mol formate.

**Figure 2:**
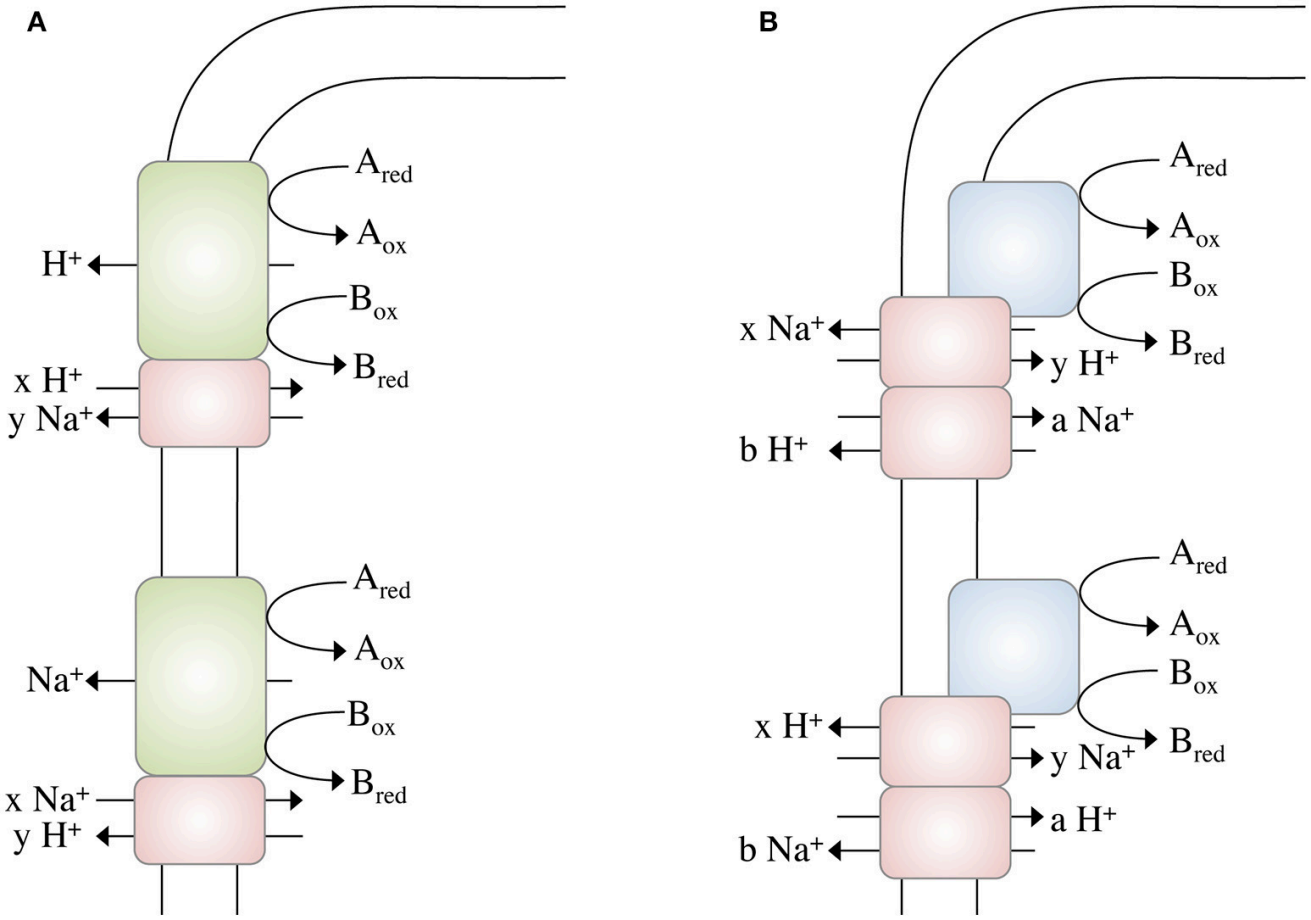
Different modes of ion gradient formation, driven by electron transfer reactions at the cytoplasmic membrane. Electron transfer from A_red_ to B_ox_ alone enables the export of a proton, which can then be exchanged into a Na^+^ gradient (or *vice versa*) by a sodium/proton antiporter **(A)**. The energy released during electron transfer from A_red_ to B_ox_ is not sufficient to export an ion (H^+^ or Na^+^) across the membrane, thus ion export is “supported” by concomitant usage of an Na^+^ or H^+^, respectively, gradient **(B)** by different antiporters. Please note that these models not only apply to electron transfer reactions but to any other (chemical) reaction that drives ion translocation (for example, methyl transfer, or decarboxylation).

These calculations bring us back to the question raised above: Could it be possible to net translocate less than one ion? This might indeed be possible by coupling two chemiosmotic enzymes operating together, but using different ion stoichiometries. Whether the membrane-bound hydrogenases (Mfh2 module) act like a classical redox loop or more like a proton pump remains to be established but the apparent lack of quinone biosynthesis genes in the genome and the similarity of the Mfh2 module to complex I of the respiratory chain is not consistent with a redox-like but rather pump-like mechanism for ion translocation. Assume the two ion translocating modules operate at different stoichiometries (Figure 2A): with one proton being “extruded” in the course of the Fdh2–Mfh2 (represented by the green box) catalyzed reaction, the connected Na^+^/H^+^ antiporter Mnh2 (represented by the pink box) can use the H^+^ potential to drive Na^+^/H^+^ antiport. If the H^+^/Na^+^ stoichiometry is greater than 1 (2 H^+^/1 Na^+^ or 3 H^+^/1 Na^+^, etc.), less than one Na^+^ is net translocated for every formate that is oxidized. This of course would require the H^+^ and Na^+^ potentials to operate at different magnitudes. The alternative is that electrogenic antiporter modules are directly hooked up to a redox module (Figure 2B). The antiporter modules have to operate in different stochiometries.

Complex I of the mitochondrial/bacterial electron transport chain has a cytosolic redox module connected to a membrane-bound, ion-translocating module. Again, the proteins of the latter are similar (although to a low extent) to multisubunit Na^+^/H^+^ antiporters and a row of four antiporters is connected by an α-helix in subunit NuoL/Nqo12 (Baradaran et al., 2013). One of the ideas is that electron transport energizes movement of the α helix that then energizes the antiporter modules. There is considerable debate whether or not complex I does translocate Na^+^ along with or instead of H^+^. However, for *Rhodothermus marinus* it was postulated that complex I indeed catalyzes Na^+^/H^+^ antiport, which is supported by the energetics of the reaction (Batista et al., 2010; Batista and Pereira, 2011; Castro et al., 2016). Complex I of *R. marinus* does not reduce ubiquinone but menaquinone (E°′ = −80 mV). The free energy change of this reaction is smaller compared to ubiquinone-reducing systems and it is discussed that simultaneous influx of Na^+^ reduces the energetic barrier for outbound H^+^ translocation. This is exactly what we propose here: the antiport activity lowers the energetic barrier for the net translocation of ions (see Figure 2B).

## Conclusion

IGP is the only mechanisms that allows living cells to metabolize low energy substrates. Among the most ancient “chemiosmotic” enzymes are bacterial and archaeal membrane-bound hydrogenases (Sapra et al., 2003; Marreiros et al., 2013), and although we have no structural information yet on them, genes coding for putative Na^+^/H^+^ antiporter are often found in their vicinity and are coexpressed (Kim et al., 2010; Lim et al., 2010), indicating that such antiporter modules are also present in membrane-bound hydrogenases. We propose that a combination of a primary pump and a secondary Na^+^/H^+^ antiporter (Figure 2A) may result in the net translocation of less than one ion. The same can be achieved by hooking up antiporter modules directly to a redox module. Net translocation of less than one ion lowers the so far accepted threshold for the minimum biological energy quantum and might explain the enigmatic growth of microbes under extreme energy limitations such as in the deep subsurface, where microbial turnover times amount to hundreds to thousands of years (Lomstein et al., 2012).

## Author contributions

VM drafted and wrote the manuscript. VH edited the manuscript and prepared the figures.

### Conflict of interest statement

The authors declare that the research was conducted in the absence of any commercial or financial relationships that could be construed as a potential conflict of interest.

## Abbreviations

SLP: substrate level phosphorylation
IGP: ion gradient-driven phosphorylation
ΔG_P_: phosphorylation potential
$\Delta {\tilde{\mu }}_{Ion}$: electrochemical ion (H^+^ or Na^+^) potential.

## References

[B1] Bae S. S. Kim Y. J. Yang S. H. Lim J. K. Jeon J. H. Lee H. S. (2006). *Thermococcus onnurineus* sp nov., a hyperthermophilic Archaeon isolated from a deep-sea hydrothermal vent area at the Pacmanus field. J. Microbiol. Biotechnol. 16, 1826–1831.

[B2] Baradaran R. Berrisford J. M. Minhas G. S. Sazanov L. A. (2013). Crystal structure of the entire respiratory complex I. Nature 494, 443–448. 10.1038/nature11871 23417064 PMC3672946

[B3] Batista A. P. Pereira M. M. (2011). Sodium influence on energy transduction by complexes I from *Escherichia coli* and *Paracoccus denitrificans*. Biochim. Biophys. Acta 1807, 286–292. 10.1016/j.bbabio.2010.12.008 21172303

[B4] Batista A. P. Fernandes A. S. Louro R. O. Steuber J. Pereira M. M. (2010). Energy conservation by *Rhodothermus marinus* respiratory complex I. Biochim. Biophys. Acta 1797, 509–515. 10.1016/j.bbabio.2010.01.020 20100453

[B5] Becher B. Müller V. Gottschalk G. (1992). N5-methyl-tetrahydromethanopterin:coenzyme M methyltransferase of Methanosarcina strain Gö1 is an Na^+^-translocating membrane protein. J. Bacteriol. 174, 7656–7660. 10.1128/jb.174.23.7656-7660.1992 1447136 PMC207478

[B6] Blaut M. Gottschalk G. (1984). Coupling of ATP synthesis and methane formation from methanol and molecular hydrogen in *Methanosarcina barkeri*. Eur. J. Biochem. 141, 217–222. 10.1111/j.1432-1033.1984.tb08178.x 6327309

[B7] Boyer P. D. (1997). The ATP synthase - a splendid molecular machine. Annu. Rev. Biochem. 66, 717–749. 10.1146/annurev.biochem.66.1.717 9242922

[B8] Brandt U. (2011). A two-state stabilization-change mechanism for proton-pumping complex I. Biochim. Biophys. Acta 1807, 1364–1369. 10.1016/j.bbabio.2011.04.006 21565159

[B9] Bücher T. Pfleiderer G. (1955). Pyruvate kinase from muscle. Methods Enzymol. 1, 435–440. 10.1016/0076-6879(55)01071-9

[B10] Buckel W. Semmler R. (1982). A biotin-dependent sodium pump: glutaconyl-CoA decarboxylase from *Acidaminococcus fermentans*. FEBS Lett. 148, 35–38. 10.1016/0014-5793(82)81237-4 6293874

[B11] Castle A. M. Macnab R. M. Shulman R. G. (1986). Measurement of intracellular sodium concentration and sodium transport in *Escherichia coli* by 23Na nuclear magnetic resonance. J. Biol. Chem. 261, 3288–3294. 3512550

[B12] Castro P. J. Silva A. F. Marreiros B. C. Batista A. P. Pereira M. M. (2016). Respiratory complex I: a dual relation with H^+^ and Na^+^? Biochim. Biophys. Acta 1857, 928–937. 10.1016/j.bbabio.2015.12.008 26711319

[B13] Dimroth P. (1982). The generation of an electrochemical gradient of sodium ions upon decarboxylation of oxaloacetate by the membrane-bound and Na^+^-activated oxaloacetate decarboxylase from *Klebsiella aerogenes*. Eur. J. Biochem. 121, 443–449. 10.1111/j.1432-1033.1982.tb05807.x 7037396

[B14] Gottschalk G. Thauer R. K. (2001). The Na^+^-translocating methyltransferase complex from methanogenic archaea. Biochim. Biophys. Acta 1505, 28–36. 10.1016/S0005-2728(00)00274-7 11248186

[B15] Hedderich R. Forzi L. (2005). Energy-converting [NiFe] hydrogenases: more than just H2 activation. J. Mol. Microbiol. Biotechnol. 10, 92–104. 10.1159/000091557 16645307

[B16] Hoehler T. M. Alperin M. J. Albert D. B. Martens C. S. (2001). Apparent minimum free energy requirements for methanogenic archaea and sulfate-reducing bacteria in an anoxic marine sediment. FEMS Microbiol. Ecol. 38, 33–41. 10.1111/j.1574-6941.2001.tb00879.x

[B17] Kashket E. R. (1982). Stoichiometry of the H^+^-ATPase of growing and resting, aerobic *Escherichia coli*. Biochemistry 21, 5534–5538. 10.1021/bi00265a024 6293545

[B18] Kim Y. J. Lee H. S. Kim E. S. Bae S. S. Lim J. K. Matsumi R. (2010). Formate-driven growth coupled with H2 production. Nature 467, 352–355. 10.1038/nature09375 20844539

[B19] Lane N. Martin W. F. (2012). The origin of membrane bioenergetics. Cell 151, 1406–1416. 10.1016/j.cell.2012.11.050 23260134

[B20] Lim J. K. Bae S. S. Kim T. W. Lee J. H. Lee H. S. Kang S. G. (2012). Thermodynamics of formate-oxidizing metabolism and implications for H2 production. Appl. Environ. Microbiol. 78, 7393–7397. 10.1128/AEM.01316-12 22885755 PMC3457120

[B21] Lim J. K. Kang S. G. Lebedinsky A. V. Lee J. H. Lee H. S. (2010). Identification of a novel class of membrane-bound [NiFe]-hydrogenases in *Thermococcus onnurineus* NA1 by *in silico* analysis. Appl. Environ. Microbiol. 76, 6286–6289. 10.1128/AEM.00123-10 20656864 PMC2937479

[B22] Lim J. K. Mayer F. Kang S. G. Müller V. (2014). Energy conservation by oxidation of formate to carbon dioxide and hydrogen via a sodium ion current in a hyperthermophilic archaeon. Proc. Natl. Acad. Sci. U.S.A. 111, 11497–11502. 10.1073/pnas.1407056111 25049407 PMC4128143

[B23] Lipmann F. (1941). Metabolic generation and utilization of phosphate bond energy, in Advances in Enzymology and Related Areas of Molecular Biology, Vol. 1, eds Nord F. F. Werkman C. H. (Hoboken, NJ: John Wiley & Sons, Inc.), 99–162. 10.1002/9780470122464.ch4

[B24] Lohmann K. (1929). Über die Phosphatfraktion im Muskel. Naturwissenschaften 17, 624–625. 10.1007/BF01506215

[B25] Lomstein B. A. Langerhuus A. T. D'Hondt S. Jørgensen B. B. Spivack A. J. (2012). Endospore abundance, microbial growth and necromass turnover in deep sub-seafloor sediment. Nature 484, 101–104. 10.1038/nature10905 22425999

[B26] Maloney P. C. (1983). Relationship between phosphorylation potential and electrochemical H^+^ gradient during glycolysis in *Streptococcus lactis*. J. Bacteriol. 153, 1461–1470. 6402498 10.1128/jb.153.3.1461-1470.1983 PMC221797

[B27] Marreiros B. C. Batista A. P. Duarte A. M. Pereira M. M. (2013). A missing link between complex I and group 4 membrane-bound [NiFe] hydrogenases. Biochim. Biophys. Acta 1827, 198–209. 10.1016/j.bbabio.2012.09.012 23000657

[B28] Martin W. Baross J. Kelley D. Russell M. J. (2008). Hydrothermal vents and the origin of life. Nat. Rev. Microbiol. 6, 805–814. 10.1038/nrmicro1991 18820700

[B29] Matthies D. Zhou W. Klyszejko A. L. Anselmi C. Yildiz Ö. Brandt K.. (2014). High-resolution structure and mechanism of an F/V-hybrid rotor ring in a Na^+^-coupled ATP synthase. Nat. Commun. 5:5286. 10.1038/ncomms6286 25381992 PMC4228694

[B30] Mayer F. Müller V. (2014). Adaptations of anaerobic archaea to life under extreme energy limitation. FEMS Microbiol. Rev. 38, 449–472. 10.1111/1574-6976.12043 24118021

[B31] Mitchell P. (2011). Chemiosmotic coupling in oxidative and photosynthetic phosphorylation. Biochim. Biophys. Acta 1807, 1507–1538. 10.1016/j.bbabio.2011.09.018 22082452

[B32] Müller V. Blaut M. Gottschalk G. (1987). Oxidation of trimethylamine to the level of formaldehyde by *Methanosarcina barkeri* is dependent on the protonmotive force. FEMS Microbiol. Lett. 43, 183–186. 10.1016/0378-1097(87)90304-1

[B33] Müller V. Winner C. Gottschalk G. (1988). Electron transport-driven sodium extrusion during methanogenesis from formaldehyde + H_2_ by *Methanosarcina barkeri*. Eur. J. Biochem. 178, 519–525. 10.1111/j.1432-1033.1988.tb14478.x 2850182

[B34] Pogoryelov D. Klyszejko A. L. Krasnoselska G. O. Heller E. M. Leone V. Langer J. D.. (2012). Engineering rotor ring stoichiometries in the ATP synthase. Proc. Natl. Acad. Sci. U.S.A. 109, E1599–E1608. 10.1073/pnas.1120027109 22628564 PMC3382517

[B35] Sapra R. Bagramyan K. Adams M. W. (2003). A simple energy-conserving system: proton reduction coupled to proton translocation. Proc. Natl. Acad. Sci. U.S.A. 100, 7545–7550. 10.1073/pnas.1331436100 12792025 PMC164623

[B36] Sazanov L. A. Hinchliffe P. (2006). Structure of the hydrophilic domain of respiratory complex I from *Thermus thermophilus*. Science 311, 1430–1436. 10.1126/science.1123809 16469879

[B37] Schäfer G. Engelhard M. Müller V. (1999). Bioenergetics of the Archaea. Microbiol. Mol. Biol. Rev. 63, 570–620. 10477309 10.1128/mmbr.63.3.570-620.1999 PMC103747

[B38] Schink B. (1997). Energetics of syntrophic cooperation in methanogenic degradation. Microbiol. Mol. Biol. Rev. 61, 262–280. 9184013 10.1128/mmbr.61.2.262-280.1997 PMC232610

[B39] Schuchmann K. Müller V. (2014). Autotrophy at the thermodynamic limit of life: a model for energy conservation in acetogenic bacteria. Nat. Rev. Microbiol. 12, 809–821. 10.1038/nrmicro3365 25383604

[B40] Slater E. C. Rosing J. Mol A. (1973). The phosphorylation potential generated by respiring mitochondria. Biochim. Biophys. Acta 292, 534–553. 10.1016/0005-2728(73)90003-0 4705444

[B41] Spahn S. Brandt K. Müller V. (2015). A low phosphorylation potential in the acetogen *Acetobacterium woodii* reflects its lifestyle at the thermodynamic edge of life. Arch. Microbiol. 197, 745–751. 10.1007/s00203-015-1107-2 25820826

[B42] Thauer R. K. Jungermann K. Decker K. (1977). Energy conservation in chemotrophic anaerobic bacteria. Bacteriol. Rev. 41, 100–180. 860983 10.1128/br.41.1.100-180.1977 PMC413997

[B43] Wallace P. G. Pedler S. M. Wallace J. C. Berry M. N. (1994). A method for the determination of the cellular phosphorylation potential and glycolytic intermediates in yeast. Anal. Biochem. 222, 404–408. 10.1006/abio.1994.1509 7864365

